# Behavioral and Psychiatric Symptoms of Dementia and Rate of Decline in Alzheimer’s Disease

**DOI:** 10.3389/fphar.2019.01062

**Published:** 2019-09-24

**Authors:** Reena T. Gottesman, Yaakov Stern

**Affiliations:** ^1^Division of Aging and Dementia, Department of Neurology, Columbia University Medical Center, New York, NY, United States; ^2^Division of Cognitive Neuroscience, Department of Neurology, Columbia University Medical Center, New York, NY, United States

**Keywords:** Alzheimer’s disease, behavioral and psychiatric symptoms, cognitive decline, functional decline, predictors of decline

## Abstract

Alzheimer’s disease causes both cognitive and non-cognitive symptoms. There is increasing evidence that the presentation and course of Alzheimer’s disease is highly heterogenous. This heterogeneity presents challenges to patients, their families, and clinicians due to the difficulty in prognosticating future symptoms and functional impairment. Behavioral and psychiatric symptoms are emerging as a significant contributor to this clinical heterogeneity. These symptoms have been linked to multiple areas of neurodegeneration, which may suggest that they are representative of network-wide dysfunction in the brain. However, current diagnostic criteria for Alzheimer’s disease focus exclusively on the cognitive aspects of disease. Behavioral and psychiatric symptoms have been found in multiple studies to be related to disease severity and to contribute to disease progression over time. A better understanding of how behavioral and psychiatric symptoms relate to cognitive aspects of Alzheimer’s disease would help to refine the models of disease and hopefully lead to improved ability to develop therapeutic options for this devastating disease.

## Introduction

Dementia is characterized by a decline in cognitive function when compared with others with similar age and education. It is an important cause of morbidity and mortality, especially in the elderly.

In 1906, Alois Alzheimer reported a case of a woman with prominent and progressive psychiatric symptoms and memory disturbance, who he followed for 5 years until her death (Maurer et al., 1997). Alzheimer’s disease is the most common cause of cognitive impairment, and its prevalence increases with age (Erkkinen et al., 2018). However, the rapidity of decline in this first patient has generally not been considered the usual course of the disease. In fact, there is significant heterogeneity in the rates and manners in which patients progress through the stages of Alzheimer’s disease (Mayeux et al., 1985).

Part of this heterogeneity is the presence of behavioral and psychiatric symptoms (BPSD). These symptoms affect over 80% of patients with AD over the course of disease, however their presentations are highly variable both between patients and over an individual’s disease course (Garre-Olmo et al., 2010a). Psychiatric symptoms can be present at all stages of disease, however specific symptoms are more common at different stages of disease. Although all symptoms worsen with disease severity, certain symptoms such as delusions, agitation, and apathy tend to become much more prevalent (Lyketsos et al., 2002).

The prevalence of psychiatric symptoms early in the course of dementia has become increasingly recognized. Mild behavioral impairment is a recently defined diagnostic construct that has been used to describe the presence of these symptoms, even in the absence clear cognitive change (Ismail et al., 2016).

Management of psychiatric symptoms is an important component of caring for these patients. Behavioral symptoms are a significant source of caregiver stress (Van Den Wijngaart et al., 2007) and contribute to the financial burden of caring for these patients (Murman et al., 2002; Schnaider Beeri et al., 2002; Herrmann N et al., 2006). These symptoms also contribute to earlier nursing home placement (Yaffe et al., 2002). A better understanding of BPSD and how they relate to neurodegenerative disease and its progression is important to patients, their families, and clinicians.

BPSD have been associated with overall clinical deterioration (Stella F et al., 2016), and there is evidence that patients with severe symptoms have identifiable neuroanatomical changes (Poulin et al., 2017). Delusions and hallucinations have been associated with atrophy within the neural networks that regulate complex behaviors (Rafii MS et al., 2014).

The ability to prognosticate clinical course is extremely important for clinicians as well as patients and their families. In addition, from a public health perspective, it is important to be able to predict costs and the health care resources needed to care for patients with AD as the population ages, as well as for identifying appropriate clinical targets for disease-modifying therapies. The trajectory of progression is not necessarily linear (Samtani et al., 2012) and is quite heterogeneous both between individuals and over the course of one case (Mayeux et al., 1985). To that end, several studies have tried to find ways of predicting clinical progression of disease. Although there are few FDA-approved treatments for BPSD at this time, the ability to predict disease course is highly valuable in its own right.

Although significant amounts of research have been devoted to a better understanding of the “negative” BPSD—namely, depression and anxiety—comparatively less has been devoted to “positive” symptoms, such as hallucinations and delusions. This review will discuss three major clusters of positive BPSD: hallucinations, delusions, and aggression/agitation. It will explore the underlying neural bases for these clusters of symptoms and discuss how these symptoms affect the rates of cognitive and functional decline in patients. A selection of the papers referenced are summarized in **Table 1**.

**Table 1:** Select papers studying BPSD and effect on decline in AD.

Author, Year	Symptom	Sample Characteristics	Analysis Modality	Findings
Wilson et al. (2006)	Hallucinations Delusional thinking Misperceptions	Rush ADRC and older adult day care centers (mean 2.2 yrs follow up) n = 568 with clinical diagnosis of AD mean 11.7 yrs education; 70.1% white; 69.2% women 478 in analysis	Linear mixed effects model for composite score of global cognition Cox proportional hazards for mortality	Psychosis at baseline: 29.6% hallucinations, 27.3% delusional thinking, 25.5% misperceptions + hallucinations and misperceptions: ↓ cognition at baseline LMM: Hallucinations: ↑ rate of decline linearly (0.20 unit/yr) and nonlinearly (0.03 unit/yr); nonlinear decline was affected by level of education Delusions: ↑ rate of decline linearly (0.08 unit/yr) Misperceptions: ↑ rate of decline linearly (0.07 unit/yr) Together: only hallucinations was associated with more rapid decline Cox: + hallucinations: 60% more likely to die; maintained when adjusting for global cognition at baseline (RR = 1.56; 95% CI 1.16-2.09); stronger in those with higher education No association for delusions/misperceptions
Connors et al. (2018)	Hallucinations Delusions	PRIME study in Australia (clinic-based, 3 yrs follow up) n = 445 with mild dementia (mean CDR-sb 5.5) 33.7% with post-secondary education; 50.1% women Psychosis at baseline: 13.5% delusions; 5.8% hallucinations; 5.4% both 34.3% without psychosis at baseline developed over 3 years	Linear mixed models for cognition, function, overall neuropsych symptoms, caregiver burden Cox proportional hazards for mortality and institutionalization	+ psychosis at baseline: ↑ CDR-sb, ↓ cognition, ↓ function, ↑ NPI, ↑ caregiver burden LMM: +delusions: ↑ disease severity (1.2 units), ↓ cognition (0.8 units), ↓ function (2.6 units), ↑ NPI (6.5 units), ↑ caregiver burden (7.8 units) + hallucinations: ↑ disease severity (0.9 units), ↓ cognition (1.0 units), ↓ function (2.7 units), ↑ NPI (4.8 units), ↑ caregiver burden (5.7 units) + both were even worse Cox: Delusions +/- hallucinations (not hallucinations alone) predicted institutionalization (HR for delusions alone 2.35 95% CI 1.48, 3.73; HR for both 4.26, 95% CI 2.31, 7.86) Neither predicted mortality
Scarmeas et al. (2005)	Hallucinations Delusions	Predictors 1&2 in USA and Europe (clinic-based, mean 4.5 yrs follow up) n = 456 with mild AD Mean MMSE 21; mean 13 yrs education; 59% women	Cox proportional hazards	Psychosis at baseline: 34% delusions, 32% hallucinations 70% developed delusions during follow up +delusions: ↑ risk of cognitive decline (RR 1.91, 95% CI 1.41-2.60), functional decline (RR 1.90, 95% CI 1.41-2.54), and institutionalization (RR 1.63, 95% CI 1.26-2.12) +hallucinations: ↑ risk of cognitive decline (RR 2.08, 95% CI 1.41-3.07), functional decline (RR 2.55, 95% CI 1.80-3.62), institutionalization (RR 1.94, 95% CI 1.40-2.70), and death (RR 1.52, 95% CI 1.08-2.15)
Hallikainen et al. (2018a)	All domains from NPI	ALSOVA cohort in Finland (clinic-based, 5 years follow up) n = 236 with very mild or mild AD (CDR 0.5-1) mean education 7.58 yrs, 51.27% female	Generalized estimating equations for disease progression Linear mixed effects model for AD severity	At baseline: 22.9% delusions, 14.8% hallucinations, 28.8% agitation By year 5: 39.7% delusions, 28.8% hallucinations, 31.5% agitation Baseline predictors of AD progression: delusions (p = 0.001), agitation (p = 0.010), aberrant motor behavior (p = 0.015), euphoria (p < 0.001) BPSD associated with AD severity over time: delusions, hallucinations, agitation, depression, anxiety, apathy, irritability, sleep disturbances, aberrant motor behavior, appetite disturbances
Barnes et al.(2009)	Hostility	Chicago Health and Aging project (population-based study, mean, 4.4 years follow-up) n = 4913 mean education 12.1 yrs, 62.2% women, 29.6% white	Mixed-effects regression model	Hostility associated with lower cognition at baseline (-0.028 units cognition for each 1 unit increase in hostility) No association between hostility and cognitive decline, including when adjusting for race and lifetime socioeconomic status
Zahodne et al. (2015)	Agitation Aggression Psychosis Depression	Predictors 1 in USA (clinic-based, 6 years follow up) n = 517 mean education 13.72 yrs, 93.2% white, 56.9% women	Latent growth curve modeling	+psychosis explained 5.3% of variance in initial cognitive impairment, 7.3% of variance in cognitive decline, 17% of variance in initial dependency, and 2.4% of variance in trajectory of dependency +agitation/aggression not related to cognitive decline or changes in dependency but 6% of variance in cognitive decline and ∼3% variance in changes in dependency were related to change in agitation/aggression, +depression explained 1.6% of variance in cognitive decline and 8.6% of variance in initial dependency
Haupt and Kurz (1993)	Delusions Angry outbursts Agitation Aggression Depression	Outpatients with mild-moderate AD in Germany over 12 months n = 66 (44 at home, 22 in institution)	Stepwise discriminant function analysis	Those admitted to nursing homes more likely to have aggressive behavior (p = 0.03) and had worse cognitive impairment (p = 0.04) Variables that contributed most to discrimination between the 2 groups: incontinence, caregiver wish to give care to someone else, cognitive decline, age, aggression, angry outbursts, depression
Peters et al.(2015)	Psychosis Agitation Aggression Affective Apathy	Incident AD from Cache County Dementia Progression Study in USA (community-based) n = 335	Kaplan-Meier plots Cox proportional hazards	50.9% with at least one neuropsychiatric symptom Predictive of progression to severe dementia: psychosis (HR = 2.007), agitation/aggression (HR = 2.946), agitation/aggression (HR = 2.946) Predictive of progression to death: psychosis (HR = 1.537), affective (HR = 1.510), agitation/aggression (HR = 1.942), at least one mild NPS (HR = 1.448), at least one NPS (HR = 1.951)
Lopez et al. (1999)	Aggression Agitation Wandering Insomnia Psychosis Depression Medication	Patients with probable AD at University of Pittsburgh (study-based, mean follow up 4.16 yrs) n = 179	Proportional hazard models	Psychosis significantly associated with decreased functional ability (RR = 2.02) and institutionalization (RR = 2.10) Adjusted for baseline age, education, MMSE, BDRS, significant associations with decreased functional ability (BDRS≥15): MMSE < 19 (RR = 4.08), psychosis (RR = 2.73), agitation (RR = 2.26), aggression (RR = 2.35), antipsychotics (RR = 1.98); psychosis associated with increased risk of institutionalization (RR = 2.11)
D’Onofrio et al. (2016)	Delusions	Patients with AD at an AD evaluation unit in Italy n = 380 mean education 5.24 yrs, 64.4% women	Comparison of means: Welch 2-sample t-test or ANOVA Wilcoxon rank sum test	Those with delusions were older, had later age of onset, worse cognitive impairment and dementia stage, more depression, higher risk of malnutrition and bedsores (p < 0.0001 for all) Those with delusions had higher NPI scores for depression (p = 0.007), hallucinations, agitation/aggression, apathy, irritability/lability, aberrant motor activity, sleep disturbances, and eating disorders (p < 0.0001 for all)
Wilson et al. (2000)	Hallucinations Delusions	Rush ADC n = 410 men education 12.0 yrs, 85.1% white, 66.8% female, mean MMSE 18.7	Random effects regression models	At baseline: +hallucinations 41.0%, +delusions 54.7% +hallucinations associated with lower cognition by 0.33 units +hallucinations: ↑ rate of cognitive decline (0.69 units/yr vs 0.47 units/yr without) +hallucinations at any point: ↑ cognitive decline (0.61 units/yr vs 0.39 units/yr without) +delusions associated with lower cognition +delusions not associated with rate of decline

## Hallucinations

### Epidemiology/Neurobiology

The reported prevalence of hallucinations in Alzheimer’s disease is wide ranging, with some estimates from 12% to 33% (Leroi et al., 2003; Wilson et al., 2006; Scarmeas et al., 2005). Unlike dementia with Lewy bodies, where visual hallucinations are a core clinical feature (McKeith et al., 2017), the hallucinations of Alzheimer’s disease can be visual, auditory (Wilson et al., 2006), olfactory, or rarely, tactile (Devanand et al., 1992). Hallucinations in Alzheimer’s disease have been associated with lower education, non-Caucasian ethnicity, and worse severity of disease (Bassiony et al., 2000;Wilson et al., 2006).

Efforts to identify the anatomy underlying visual hallucinations have yielded variable results. Hallucinations have been associated with occipital atrophy (Holroyd et al., 2000) and hypoperfusion in left dorsolateral prefrontal, left medial temporal, and right parietal cortices (Lopez et al., 2001). One study found that atrophy in the right supramarginal gyrus predicted worsened hallucinations over 3 years (Donovan et al., 2014). It would be intuitive to propose that more global network dysfunction underlies the relationship between hallucinations and cognitive decline. However, there have been few formal studies assessing this network. The right anterior insula has been proposed as the “core region” for hallucinations, in part, due to its role in integrating external sensory input with the internal milieu (Blanc et al., 2014).

Deficiency in acetylcholine from the basal forebrain underlies attentional and arousal deficits in Alzheimer’s disease and other neurodegenerative diseases (Pepeu et al., 2013). Accordingly, acetylcholinerase inhibitors have long been a mainstay in the treatment of Alzheimer’s disease. It has been proposed that a form of cholinergic deficiency syndrome, which is characterized by restlessness, memory disturbances, and visual hallucinations, and was initially described as an iatrogenic syndrome from anticholinergic treatment, may exist in a more chronic form in neurodegenerative diseases (Lemstra et al., 2003). Although this proposal suggested that cholinergic deficiency syndrome is more specific to dementia with Lewy bodies, the symptoms of the syndrome are often present in patients with Alzheimer’s disease as well. An EEG study of patients with Alzheimer’s disease or dementia with Lewy bodies found that patients with hallucinations and Alzheimer’s disease had similar slowing of EEG activity as those with hallucinations and dementia with Lewy bodies, suggestive of cholinergic loss in both populations (Dauwan et al., 2018).

The effect of hallucinations on cognition has been associated with genetic predispositions. A large study using 900 autopsy-confirmed cases of AD from the National Alzheimer’s Coordinating Center (NACC) data to study the effect of psychosis and *APO* ε4 on cognition found that hallucinations were significantly associated with worse cognition, and that the presence of *APO* ε4 attenuated this relationship (Qian W et al., 2018). Interestingly, the presence of *APO* ε4 was also significantly associated with more Lewy body pathology in this study.

Additionally, hallucinations have been associated with sleep disturbances. It has been hypothesized that this is due to dysregulation of the neurotransmitter systems involved in sleep (Sinforiani et al., 2007). Hallucinations tend to be more likely to occur during sleep or during sleep–wake phase transitional phases (i.e. falling asleep and waking up) (Sinforiani et al., 2007). Intuitively, this supports an association between the sleep–wake cycle and the presence of hallucinations.

### Contribution to Rates of Decline

Hallucinations are associated with more severe cognitive impairment (Wadsworth et al., 2012), are persistent (Holtzer et al., 2003), and may increase in incidence over time (Vilalta-Franch et al., 2013). There have been several studies both in community-based and clinic-based cohorts that assessed the relationship between hallucinations and the rate of decline. They have largely shown that the presence of hallucinations at baseline is associated with more rapid decline. Wilson et al. (2006) followed patients for an average of 2.2 years in the Rush Alzheimer’s Disease Center and community-dwelling adults and found that in addition to being related to poorer performance on cognitive screening (average MMSE of 10.7 for patients with hallucinations versus 14.1 for those without), patients demonstrated more rapid cognitive decline if they had hallucinations at baseline. Patients with hallucinations also had increased risk of mortality by the end of the study (RR, 1.55). Similarly, Connors et al. (2018) demonstrated that patients in memory clinics in Australia with hallucinations had worsened dementia severity, lower cognition and function, and greater caregiver burden over a 3-year period. Similar results have been found by several other studies (Forstl et al., 1993; Vilalta-Franch et al., 2013; Tchalla et al., 2018).

A limitation of any longitudinal study of hallucinations is that symptoms fluctuate over time (Devanand, 1999). Therefore, extended follow-up would be beneficial and contribute to improved robustness of the analysis. Hallikeinen et al. (2018a) analyzed data from the ALSOVA study, a cohort of patients with very mild and mild AD (CDR 0.5-1 at baseline) in Finland, using generalized estimated equations (GEE), and did not find evidence that hallucinations predicted disease severity over 5 years. However, using linear mixed models, they did find that hallucinations were significantly associated with Alzheimer’s disease severity over time.

In contrast, Scarmeas et al. (2005) analyzed data from participants in the Predictors 1 and Predictors 2 cohorts, which are longitudinal studies of patients diagnosed with probable Alzheimer’s disease in multiple centers in the United States and Europe (Stern et al., 1993; Scarmeas et al., 2004), for an average of 4.5 years, and up to 14 years, and using Cox analysis looked at the risk of reaching specified functional and cognitive endpoints. They found that the presence of hallucinations was associated with increased risk of cognitive (RR, 1.62) and functional (RR, 2.25) decline, institutionalization (RR, 1.60), and death (RR, 1.49). It is possible that part of the difference in results may be related to the use of different statistical measures.

An additional limitation of studying the role of hallucinations in Alzheimer’s disease is that it is often difficult to determine whether there is comorbid dementia with Lewy bodies (DLB). One large study found evidence of Lewy Body pathology in the brains of 60.7% of a cohort of clinically diagnosed Alzheimer’s disease. When NIA-RI criteria, which require AD pathology to make the diagnosis (The National Institute on Aging, and Reagan Institute Working Group on Diagnostic Criteria for the Neuropathological Assessment of Alzheimer’s Disease, 1997), were applied, Lewy bodies were found in 56.8% of the cohort (Hamilton, 2000). Furthermore, it can be clinically challenging to discriminate between the two conditions. Chung et al. found that pathology-confirmed AD did have distinct clinical phenotypes when co-occurring with Lewy body pathology (Chung et al., 2015). In contrast, Roudil et al. found that there were no significant clinical differences in many clinical and neuropsychological aspects between pathology-confirmed AD with Lewy bodies (either confined to the amygdala or more widespread in the cortex) and without, including the presence of hallucinations (Roudil et al., 2018), although this was a much smaller study and used a cohort that may have been worse cognitively.

## Aggression/Agitation

### Epidemiology/Neurobiology

Agitation and aggression are common in Alzheimer’s disease, with one large study of electronic health records estimating the prevalence of agitation as over 50% in mild cases (Halpern et al., 2019), as measured in a large study of electronic health records. It may be among the most significant contributors to longitudinal caregiver stress, possibly due to undermining a caregiver’s sense of security (Hallikainen et al., 2018b). However, there are fewer studies of agitation and aggression compared with other BPSD (Victoroff et al., 2018). One study of pathologically confirmed AD with intermediate or high pathology load found that patients with agitation and aggression tend to do worse on cognitive testing and are worse functionally (Sennik et al., 2017). It is a significant symptom from a public health perspective due to its association with higher healthcare costs through increased institutionalization (Costa et al., 2018) as well as “informal costs,” such as caregiver time (Rattinger et al., 2019).

Like hallucinations, the presence of agitation has been correlated with multiple anatomic locations. Agitation has been correlated with increased neurofibrillary tangle burden in the orbitofrontal and anterior cingulate cortices (Tekin et al., 2001). Supporting this is an anatomical study using ADNI data which found that the presence of worsening agitation and aggression was associated with greater atrophy in frontal, insular, amygdala, cingulate, and hippocampal regions of interest in patients with mild cognitive impairment and AD dementia over 2 years (Trzepacz et al., 2013). It has also been suggested that right frontal lobe dysfunction may be predominant (Lopez et al., 2001). Functional imaging studies have similarly suggested that agitation is associated with lower metabolism in frontal and temporal regions (Sultzer et al., 1995). Interestingly, Ehrenberg et al. (Ehrenberg et al., 2018) found that agitation was associated with neurofibrillary tangle pathology at Braak stages I to IV but not at levels V to VI, suggesting that subcortical pathology may be an important contributor as well.

Aggression has been linked to increased amyloid burden. Transgenic mice expressing a human APP mutation have been shown to be significantly more aggressive than non-transgenic littermates, even early on the course of disease (Alexander et al., 2011). In humans, a large study found that the presence of agitation/aggression was directly correlated to high burden of amyloid pathology in a cohort of pathologically confirmed AD (Sennik et al., 2017), and that other clinical diagnoses were attributed to patients with agitation/aggression, including dementia with Lewy Bodies and frontotemporal dementia. In this cohort, phosphorylated TDP-43 deposits were more common in male patients with agitation, indicating the difficulty in diagnosing patients appropriately when behavioral disturbances are present.

In a cohort of pathologically confirmed Alzheimer’s disease, the presence of a higher overall score on the Cohen-Mansfield Agitation Inventory (Cohen-Mansfield, 1986), a scale specifically for agitation and aggression in the elderly, was significantly associated with decreased levels of 5-HIAA in the hippocampus. In this cohort, the presence of physically nonaggressive behavior (which may still be distressing to the patient and caregiver) was significantly associated with increased dopamine catabolism in the cerebellum (Vermeiren et al., 2014). An autopsy-based study of individuals with a clinical diagnosis of Alzheimer’s disease showed that aggression was a significant predictor of decreased cholinergic innervation on autopsy, and overactivity was the best predictor of decreased serotonergic innervation (Garcia-Alloza et al., 2005).

### Association With Rates of Decline

Agitation, alone and in combination with other BPSD, has been associated with disease severity over time, but inconsistently with disease progression. There are few studies that assess agitation as an isolated factor. Haupt and Kurz (Haupt and Kurz, 1993), in a small clinic-based study, found that aggression was one factor that predicted institutionalization over 1 year. Similarly, Peters et al. (2015) in the Cache County Dementia Progression Study, found that agitation/aggression was a significant predictor of the risks of both of severe dementia (defined as CDR ≥ 2 or MMSE ≤ 10) and death (HR 2.946, 1.942 respectively) in a Cox proportional hazards model, although the baseline status of participants in that cohort is unclear. Further, Lopez et al. (1999) in their study of the effects of psychiatric symptoms and psychiatric medications on disease progression found that the presence of either aggression or agitation was independently associated with a significantly increased risk of shorter time to significant functional impairment (RR, 2.35, 2.26, respectively) while controlling for age, education, sex, and baseline cognitive and functional status.

In contrast, Barnes and colleagues, using data from the Chicago Health and Aging Project, a longitudinal population-based study, found that hostility was both associated with worse cognition at baseline in both non-Hispanic whites and African Americans but not with cognitive decline over 4.4 years, in a mixed-effects regression model (Barnes et al., 2009). Zahodne et al. studied data from the Predictors 1 cohort and found that agitation/aggression at baseline were not correlated with cognition at baseline, functional decline, or cognitive decline in a latent growth curve model over 6 years. However, change over time in agitation/aggression did account for a small amount of the variability in cognitive decline over time (Zahodne et al., 2015). Similarly, in the ALSOVA study, agitation at the time of diagnosis did not predict disease progression but was significantly associated with severity of disease over 5 years (Hallikainen et al., 2018a).

Of note, Hallikainen et al. found that, along with agitation and aggression, aberrant motor behavior tended to track with disease severity and was a predictor of disease progression (Hallikainen et al., 2018a). Aberrant motor behaviors are sometimes, (Aalten et al., 2003; van der Linde et al., 2014) but not always, (Garre-Olmo et al., 2010b) clustered together with agitation in studies that have looked for grouping of neuropsychiatric symptoms out of the neuropsychiatric inventory (NPI) by methods such as factor analysis and latent class analysis.

## Delusions

### Epidemiology/Neurobiology

Delusions are a well-recognized symptom of Alzheimer’s disease although Holtzer et al. found that they may worsen initially and then become less prevalent over time (Holtzer R et al., 2003), which can make estimating its prevalence difficult. Delusions tend to be considered either “persecutory” or related to misidentification phenomena, such as Capgras syndrome or phantom boarder syndrome. The presence of delusions tends to be combined with the presence of hallucinations to create the construct of psychosis. Although psychosis certainly encompasses both of these symptoms, delusions may be representative of different neural circuits than hallucinations. Clinically as well, it is worthwhile considering the two symptoms separately. There are many circumstances where visual and auditory hallucinations may occur in the absence of fixed beliefs on the part of the patient believing that they are true, and delusions can occur in the absence of hallucinations. Interestingly, delusions may have a different impact on patients’ functioning than hallucinations or agitation. Bertrand and colleagues (Bertrand et al., 2017) found that patients with mild to moderate AD who had delusions had a decreased ability to express treatment choice preference, potentially impacting their ability to consent to medical care. In contrast, patients with hallucinations, agitation/aggression, and several other BPSD did not have weaknesses in decision-making abilities.

Structurally, the presence of delusions has been associated with decreased gray matter density in the right inferior frontal gyrus and inferior parietal lobule, as well as the left inferior and medial frontal gyri and claustrum in patients with mild AD (Bruen et al., 2008). Interestingly, Fischer and colleagues compared MRI scans in patients with MCI before and after the onset of delusions (generally within the span of 6 months), and found significant differences in gray matter morphology in 14 locations, including the bilateral insulae, the cerebellum, the right thalamus and posterior cingulate gyrus, and the left precuneus, left superior temporal gyrus, and parahippocampal gyrus (Fischer et al., 2016). During that time, some patients had converted from MCI to mild AD, and the mean cognition had worsened as well. The presence of delusions has also been associated with abnormalities in the integrity of white matter in the left parietooccipital region and the corpus callosum (Nakaaki et al., 2013), as well as advanced neurofibrillary tangle pathology (Ehrenberg et al., 2018).

### Association With Rates of Decline

Delusions are often studied in conjunction with hallucinations. Their presence is often associated with poorer performance on cognitive testing cross-sectionally (Jeste et al., 1992) as well as faster decline longitudinally. Connors et al, in the PRIME study in Australia, found that the presence of delusions alone was associated with worse cognition, function, and dementia severity, as well as increased caregiver burden, over 3 years using a linear mixed model. Delusions also predicted institutionalization, but not mortality (Connors et al., 2018). Similarly, Scarmeas et al. found in the Predictors cohort that delusions were associated with increased risk for cognitive (RR, 1.50) and functional (RR, 1.41) decline as well as institutionalization (RR, 1.60) and mortality (RR, 1.49) (Scarmeas et al., 2005).

Interestingly, D’Onofrio and colleagues, in a single-center study, found that delusions were associated with a trend toward significantly longer disease duration in patients with mild to moderate AD (D’Onofrio et al., 2016). Further, Wilson et al. studied the effects of hallucinations and delusions over 4 years and found that delusions were not associated with the rate of cognitive decline (Wilson et al., 2000). There are few studies that separate delusions from hallucinations in longitudinal studies; however, the evidence regarding the effect of delusions on the rate of cognitive decline seems to be less conclusive. Additional studies would be useful in better understanding the effect of the presence of delusions.

## Using BPSD to Inform Models of Disease

The clinical diagnosis of probable Alzheimer’s disease requires the presence of worsening cognition in either an amnestic or nonamnestic pattern (McKhann et al., 2011). Noncognitive symptoms are not considered in these diagnostic criteria; however, BPSD are a prevalent aspect of disease. Although the time point of disease during which these symptoms initially manifest varies, they persist over time and increase with worsening of disease. These symptoms may be more challenging for families to address than cognitive symptoms and represent a public health concern due to the increased morbidity, mortality, and associated healthcare costs. The areas of the brain which correlate to these symptoms are likely affected due to the spread of pathology across neural networks, and it remains unclear why these behavioral areas become affected early in some patients. The genetic and environmental contributors to the presence and timing of these symptoms have yet to be elucidated. The variable results in attempting to identify specific brain regions associated with specific symptoms suggests that the presence of BPSD may be reflective of network-wide dysfunctions. Indeed, the presence of hyperactivity, which includes agitation, has been linked to changes in the anterior salience network, which is important in generating appropriate responses to the external environment, in resting-state functional MRI (Balthazar et al., 2014). It is worth considering whether the presence of these symptoms should be considered as important in diagnosing Alzheimer’s disease as are cognitive changes. Due to the extensive heterogeneity in Alzheimer’s disease, different models of disease prediction may need to be developed. In fact, Razlighi et al, in developing and validating a longitudinal Grade of Membership (L-GoM) model to predict time to institutionalization, full-time care, and death in the Predictors 1 and 2 cohorts, included the presence of BPSD, such as psychosis and wandering (Razlighi et al., 2014). Further studies are needed to better understand these issues, so that ultimately effective symptomatic treatments can be developed.

Additionally, the relationship between neuropathological changes and BPSD is likely affected by outside factors. Casanova et al, in their review of clinicopathological correlates of BPSD, note that neuroleptics may increase the risk for cerebrovascular events in patients with dementia which may itself increase the risk for certain BPSD (namely, depression and apathy) and also worsen cognitive decline (Casanova et al., 2011). Neuroleptics and other antipsychotics may also have an impact on disease presentation and progression; these medications may reduce symptoms of BPSD, although a consensus of geriatric mental health experts noted that the evidence is limited and inconsistent with regard to agitation and aggression (Salzman et al., 2008). However, Lopez et al. (1999) found that antipsychotics were associated with increased functional decline, and hypothesized that this effect could be related to sedation or extrapyramidal effects. Nonpharmacologic therapies such as music, bright light, and pet therapy have also been noted to be possibly effective in reducing symptoms of BPSD, although such therapies are often part of a broader care program (Forlenza et al., 2017; Doody et al., 2001) and can be difficult to isolate for purposes of a trial. Many, but not all, studies looking at the effect of BPSD on disease progression take pharmacologic therapy into account; however, we were unable to find studies that accounted for nonpharmacologic approaches. If the use of nonpharmacologic therapies can be strengthened with additional clinical trials, this will need to be taken into account in future studies of BPSD as well.

Furthermore, the use of cholinesterase inhibitors and memantine may confound these effects as well; in the Predictors 2 cohort, cholinesterase inhibitors were associated with delayed functional decline, and memantine was associated with delayed time to death when controlled for several patient characteristics, including the presence of psychiatric symptoms (Zhu et al., 2013). Although this does not confer causality, it implies that current standard of care treatment for Alzheimer’s disease may affect the influence of BPSD on dementia severity.

An additional factor not usually accounted for is the role of social support or family involvement in care, whose relative absence has been associated with increased depression in the elderly (Sonnenberg et al., 2013). In contrast, Chan et al. found that those with a child or child-in-law as the caregiver were more likely to be reported as having psychopathy, although it is not clear whether this was due to a difference in prevalence of the symptoms or a difference in likelihood of reporting them (Chan et al., 2003). It is plausible that the presence of a more extensive social support network and family presence could help to alleviate distress caused by symptoms, such as hallucinations and perhaps mitigate the effect of these symptoms on functioning and cognitive decline. Further studies would be useful in clarifying this effect.

An important limitation in many studies has been the lack of pathologic confirmation of diagnosis. BPSD are present in many neurodegenerative diseases, such as Lewy body disease and frontotemporal dementia. Clinically, these entities can be difficult to distinguish from each other, particularly early in the course of disease. As an example, the frontal variant of Alzheimer’s disease may be misdiagnosed as frontotemporal dementia—one clinic-based study found that nearly 40% of cases diagnosed clinically as frontotemporal dementia were changed to a diagnosis of Alzheimer’s disease based on PET scan results (Ossenkoppele et al., 2013). Similarly, an autopsy series of Lewy body disease, Alzheimer’s disease, and AD with amygdala-predominant Lewy bodies found that the presence of visual hallucinations did not distinguish between the groups, although they tended to occur earlier in Lewy body disease. The underlying pathologies of Alzheimer’s disease and Lewy body disease frequently coexist (Hamilton, 2000), which can make it difficult to ascertain clinically which pathology is causing the symptoms.

Additionally, there is no standard method for estimating baseline effects on downstream events. Statistical methods that are commonly used are Cox proportional hazards, latent growth curve modeling, and generalized estimating equations. Each model has its strengths; however, it can be difficult to directly compare results obtained from different methods, leading to additional difficulty in using baseline characteristics to predict effects over time.

In the clinical setting, the presence of early hallucinations and agitation may direct the clinician’s diagnostic approach away from the possibility of underlying Alzheimer’s pathology. Current diagnostic criteria for other neurodegenerative disorders encourage this approach; however, this may be an incomplete understanding. It may be more useful to view the presence of BPSD in the setting of other cognitive changes as a relatively nonspecific symptom of underlying neural network dysfunction. Biomarker-based diagnosis in the form of imaging, as well as CSF and serum diagnostics, is likely to be increasingly important in refining clinical diagnostic criteria.

## Conclusion

The ability to prognosticate is critically important for patients and their families. The balance of the evidence suggests that early presence of positive BPSD may predict faster progression of disease. This does not account for the effect of negative BPSD (namely, depression and apathy, which likely also contribute to progression of disease). Although the etiology underlying this effect is not well understood, it is likely a complex combination of genetic predisposition and comorbid neuropathology. Cohort studies that followed patients with early dementia have been vitally important in elucidating this effect, although few studies have followed patients for long enough to capture the variability over a long disease course. Additional long-term studies are needed to ensure the generalizability of these effects.

## Author Contributions

RG and YS contributed to manuscript preparation and editing.

## Funding

This work was supported by R01 AG007370 from the NIA and by 5T32 NS007153 from the NINDS (Elkind, PI).

## Conflict of Interest Statement

The authors declare that the research was conducted in the absence of any commercial or financial relationships that could be construed as a potential conflict of interest
